# Narrative Review on Infants’ Thermoregulatory Response to Heat

**DOI:** 10.3390/ijerph22081265

**Published:** 2025-08-13

**Authors:** Eline van de Kamp, Hein Daanen

**Affiliations:** Department of Human Movement Sciences, Vrije Universiteit Amsterdam, 1081 HV Amsterdam, The Netherlands; h.a.m.daanen@vu.nl

**Keywords:** thermoregulation, infants, heat, health risks, extreme temperatures, surface area-to-mass ratio, skin blood flow, sweating, skin temperature, core temperature

## Abstract

Infants are at a higher risk of heat-related morbidity and mortality compared to children and adults. However, it remains unclear whether this vulnerability stems from immature thermoregulatory mechanisms or simply from their dependence on caregivers. This narrative review examines current literature on infant thermoregulation during heat exposure and explores how unique physiological characteristics may influence vulnerability. Key differences in infants compared to older individuals include their larger surface area-to-mass ratio, which (1) facilitates heat dissipation when skin temperature exceeds ambient temperature, but compromises heat loss in reversed conditions, and (2) likely enables a large portion of an infant’s blood volume to shift to the skin, promoting heat loss but reducing blood volume in the central circulation. Infants also have a relatively high metabolic heat production. Additionally, their lower sweat output per gland may represent either a limitation or a different thermoregulatory strategy. Contrary to common assumptions, most components of infants’ thermoregulatory system do not appear inherently immature; rather, their distinct physiological characteristics—combined with their reliance on caregivers—shape how and when heat exposure may become harmful. Nevertheless, further research is needed to better understand how these interacting factors influence infants’ ability to maintain stable core temperature. Meanwhile, coordinated efforts by caregivers, health professionals, and policymakers are essential to minimize infants’ heat-related health risks.

## 1. Introduction

Infants, defined as those under the age of one, are widely recognized as a vulnerable population that is at a higher risk of adverse health effects and mortality during extreme heat. Several studies have documented increased mortality rates during heat exposure within this age group. For instance, Basagaña et al. [1] observed a 25% increase in infant mortality on extremely hot days in Spain. Basu and Ostro [2] estimated that a 4.7 °C (10 °F) rise in daily temperature is associated with a 4.9% rise in infant mortality in California, the largest increase among all age groups. In France, Fouillet et al. [3] reported an excess mortality of 29% among male infants during the 2003 heatwave. Scovronick et al. [4] found that in South Africa, the relative risk of mortality associated with extreme temperatures was highest for children under 5 years old, with a relative risk of 1.24. Geruso and Spears [5] analyzed data from Demographic and Health Surveys from over 50 countries in Latin America, Asia, and Africa, and found a significant impact of extremely hot and humid days on infant mortality, particularly during the first month of life.

In addition to mortality, extreme heat also poses significant risks for infant morbidity. A review by Lakhoo et al. [6] showed that high temperatures were associated with hand, foot, and mouth disease and hospital admissions in infants. Mannan et al. [7] identified a positive relation between ambient temperature (T_a_), the heat-humidity index, and the occurrence of severe diseases in infants living in two regions of India. Moreover, Teyton et al. [8] found that heatwaves led to a rise in all-cause emergency visits among infants in California.

The vulnerability of infants to heat-related mortality and morbidity can, at least partly, be attributed to their dependence on caregivers. Infants cannot seek a cooler environment, drink more water, or remove excess clothing on their own, and rely on crying to express discomfort [9]. Caregivers often estimate the thermal state of an infant based on their own, which may lead to incorrect assumptions [10]. Failure of caregivers to provide adequate fluid intake during heatwaves was found to be a contributing factor in heat-related illness among hospitalized infants [11]. Additionally, pediatric vehicular heatstroke has become a significant cause of mortality. Since 1998, over 987 children in the United States have died of heatstroke after being left unattended in a car, with 31% of deaths under the age of one [12].

Besides their dependency on caregivers, infants might also be more vulnerable to heat due to their thermoregulation. Thermoregulation refers to the process by which the body maintains its internal temperature within a safe range regardless of changes in the external environment [13]. The body produces heat through metabolic processes, and heat exchange with the environment happens through evaporation, radiation, conduction, and convection. The main non-behavioral thermoregulatory mechanisms during heat are cardiovascular and vasomotor responses, which increase skin blood flow, and the production and evaporation of sweat [14,15].

There are different views in the literature regarding the maturity of thermoregulatory mechanisms in children, including in infants. Several studies suggest that these mechanisms are still immature in children, which makes them more vulnerable to extreme temperatures. Tsuzuki [16] states that this immaturity in young children limits their ability to effectively regulate body temperature. Inoue et al. [17] note that the sweat mechanism in children is underdeveloped in the prepubertal phase. Williams [18], Xu et al. [19], and Lakhoo et al. [6] also emphasize that developmental differences in thermoregulation play a key role in children’s vulnerability during heat exposure.

On the other hand, some researchers challenge the idea that thermoregulation in children is inherently immature or underdeveloped. Smith [20] notes that there is accumulating evidence of limited differences between children and adults in thermoregulation during moderate heat. Singer [21] states that considering children’s thermoregulation as deficient is one of the most common misconceptions in thermal balance, and only holds true for preterm infants. Falk and Dotan [22] revisited some widely accepted child-adult differences and concluded that “children are likely not as disadvantaged in terms of thermoregulation as they have long been portrayed to be”.

However, most existing research has focused on older children, with conclusions often extended to infants. Yet, the first few years of life involve rapid growth and development, meaning findings may not be directly transferable. Given that infants experience higher heat-related health risks—alongside rising global temperatures and more frequent and intense heatwaves [23]—it becomes increasingly important to examine thermoregulation specifically in this age group.

Therefore, this narrative review aims to clarify what is known, identify critical gaps, and inform future research and public health strategies. To achieve this, it brings together findings from the limited number of studies specifically focusing on infant thermoregulation during heat exposure, examines studies on thermoregulation in children (>1 year) to evaluate which findings might apply to infants, and explores how specific infant characteristics may affect vulnerability. Given the scarcity of studies on infant thermoregulation in heat, a narrative review was found more suitable than a systematic review. The current review argues that infant thermoregulation is not inherently immature, but instead marked by distinct physiological characteristics which—together with their reliance on caregivers—can increase vulnerability during heat exposure under certain conditions.

## 2. Differences in Thermoregulation Between Infants and Older Individuals

### 2.1. Differences in Surface Area-to-Mass Ratio

One of the most noticeable differences between infants and adults is their surface area-to-mass ratio. The body’s surface area, primarily the skin, plays a crucial role in heat exchange with the environment. Relative to their body mass, infants have a significantly greater body surface area (BSA) compared to adults.

Using Mosteller’s [24] formula for BSA, which gives accurate values for infants as well as adults [25] and aligns well with BSA measurements of 3D scans of infants [26], we calculate BSA as follows:

$$BSA=\sqrt{\frac{weight\left(kg\right)\times height(cm)}{3600}}$$

**Figure 1:**
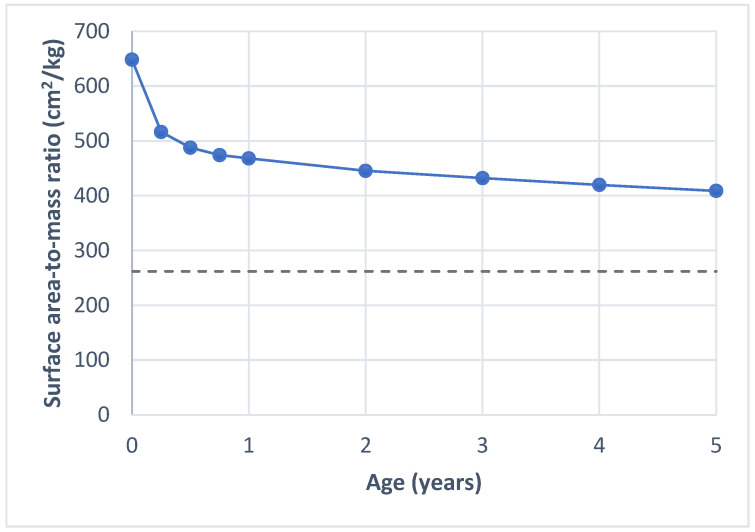
Surface area-to-mass ratio by age. The solid line shows the values calculated for children using the formula for body surface area by Mosteller [24] and child growth standards for height and weight by WHO [27]. The dashed line shows the average surface area-to-mass ratio for adults, based on values reported by Verbraecken et al. [28].

Applying the WHO child growth standards for height and weight [27], we find that a newborn has the highest surface area-to-mass ratio, with an average BSA of 648 cm^2^ per kg. This ratio declines steeply during the first year of life, falling to 468 cm^2^ per kg by the age of one—representing a 28% decrease. This trend of decreasing surface area-to-mass ratio continues as the child grows (Figure 1).

A greater surface area-to-mass ratio is an advantage in dry heat loss (convection, radiation, conduction) when the ambient temperature (T_a_) is below skin temperature (T_sk_), and for wet heat loss, as there is a relatively larger BSA for sweat evaporation [29]. Under these conditions, a larger proportion of heat could be dissipated through the skin. Davies [30] demonstrated that children, due to their larger surface area-to-mass ratio, lose a larger proportion of heat through convection and radiation during heat exposure compared to adults. More recent studies have further explored the role of BSA and found that a lower surface area-to-mass ratio is associated with an increased risk of heat intolerance in adults [31,32].

In extreme heat, when T_a_ exceeds T_sk_, the advantage of a large surface area-to-mass ratio is lost as the heat flux turns the other way. This leads to dry heat gain from the environment. However, as stated by Rowland [33], whether this actually increases core temperature remains unclear. While not focused directly on infants, some studies have shown no significant differences in the increase in rectal temperature (T_rec_) between children and adults in extreme heat (with T_a_ ranging from 41 °C to 48 °C) [34,35,36]. Delamarche et al. [36] did note that while the increase in T_rec_ at rest at 45 °C was similar to adults, children experienced greater convective and radiative heat gains. This suggests that their higher surface area-to-mass ratio does lead to more heat absorption; however, the lack of a significant difference in T_rec_ may be due to other mechanisms at play, such as heat dissipation through the evaporation of sweat.

Thus, while infants’ higher surface area-to-mass ratio can facilitate heat loss in moderate heat, it may contribute to heat gain in extreme temperatures—though it remains unclear whether this is balanced out by other mechanisms such as the evaporation of sweat.

### 2.2. Differences in Metabolic Rate

**Figure 2:**
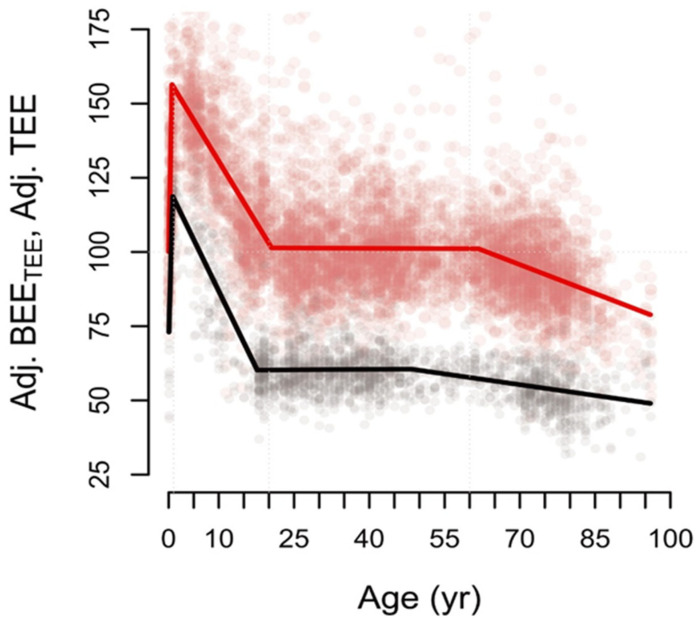
Size-adjusted total (red) and basal (black) energy expenditure over the life course. Energy expenditures are expressed as percentages of adult-level adjusted total energy expenditure (~100%). BEE: basal energy expenditure. TEE: total energy expenditure. From Pontzer et al., Daily energy expenditure through the human life course. *Science* 373, 808–812 (2021). Reprinted with permission from AAAS [37].

The metabolic rate equals the amount of heat produced by the body in the absence of external work. A recent analysis by Pontzer et al. [37] investigated metabolic rates throughout the course of life, specifically focusing on total and basal energy expenditure (Figure 2). The study found that newborns, during the first month of life, have energy expenditures comparable to those of adults when adjusted for fat-free mass. However, this increases rapidly during the first year of life, reaching its peak around 8–9 months of age. From birth to this peak, energy expenditure rises about 85%. From 9 to 15 months of age, energy expenditure is past its peak but still approximately 50% higher than in adults. After the first year of life, this steadily declines until reaching stable levels at around 20 years of age. These findings indicate that infancy is characterized by a high size-adjusted energy expenditure when compared to other stages of life. These increased expenditures likely reflect the increased metabolic demands associated with growth and development [38].

Thus, metabolic heat production peaks around 8–9 months of age. At this stage, infants must dissipate more heat per unit of surface area than both newborns and older children to maintain thermal balance. Earlier studies showed similar trends of increasing metabolic heat production during the first year of life. Bauer et al. [39] observed an increase in resting energy expenditure when comparing the first week to the sixth week of life. Azaz et al. [40] showed that during the first three months of life, infants started to produce increasingly more heat per unit of BSA during sleep.

### 2.3. Differences in Cardiovascular Response

The cardiovascular system plays an important role in heat dissipation during heat stress. By adjusting the heart rate and stroke volume, cardiac output increases, and by adapting vasomotor responses, cutaneous vasodilation occurs, together resulting in more blood flow to the skin [41,42]. This facilitates heat loss to the environment when T_a_ is below T_sk_. Already in newborns, thermal stimuli can trigger increases in heart rate and skin blood flow, demonstrating that their cardiovascular system already responds to heat [43,44,45].

Heart rate decreases with age; therefore, infants have higher heart rates compared to older individuals [46,47]. An increase in core temperature results in a rise in heart rate, as the body needs to compensate for decreased blood flow to the heart while maintaining nutritional blood flow to the tissues [48]. The extent of heart rate rise associated with increasing temperature is age-dependent as well, where both the relative and absolute increase is most pronounced in infants [49,50].

Studies on skin blood flow responses to heat have shown that children exhibit higher skin blood flow compared to adults. Martin et al. [51] looked at maximal skin vascular conductance by measuring skin blood flow on the forearm at a T_sk_ of 42 °C in participants aged 5 to 84 years, and found that the youngest participants had the highest skin blood flow. Similarly, Falk et al. [52] observed that forearm blood flow during exercise at 42 °C was higher in prepubertal boys than in late pubertal boys.

There is a lack of studies investigating skin blood flow in infants and comparing this to older children and adults under the same thermal conditions. However, if these age-related trends extend to infants as well, they would have an advantage in dry heat loss through convection and radiation when T_a_ is below T_sk_, but a disadvantage when T_a_ is above T_sk_, as heat would be absorbed rather than dissipated.

Additionally, the higher skin blood flow and greater increase in heart rate in response to heat in younger individuals may indicate challenges in maintaining adequate venous return. As noted by Drinkwater et al. [34], due to the larger surface area-to-mass ratio in children, a larger proportion of their total blood volume may need to be diverted to the skin. This would result in less blood flow to internal organs and muscles, potentially leading to heat intolerance. Both Drinkwater et al. [34] and Falk et al. [52] observed that younger children showed more signs of headaches, fatigue, and dizziness during heat exposure, likely due to the redistribution of blood volume from central to peripheral circulation.

Thus, infants might also have relatively high skin blood flow during heat, which facilitates heat dissipation to the environment when T_a_ is below T_sk_. However, the greater increase in heart rate in infants as a response to high temperatures suggests that their cardiovascular system needs to work harder to compensate for decreased blood flow to the heart, potentially leading to symptoms of heat intolerance. More research on infants specifically is needed to draw conclusions regarding cardiovascular limitations during heat.

### 2.4. Differences in Sweat Production

Heat loss through evaporation—also known as wet heat loss—occurs primarily through sweating, as opposed to wet heat loss from the respiratory airways. Eccrine sweat glands start developing around 14 weeks of gestation, and by 28 weeks of gestation, all sweat glands are formed [53]. No new sweat glands are formed after birth [53,54]. As a result, the density of sweat glands per unit of surface area is highest in newborns. Infants in the first two weeks of life have an average of 414 eccrine sweat glands per cm^2^ of skin on the thigh, which is 6.5 times the density found in adults [55].

Around 36 weeks of gestation, the sweat glands become active. This means term infants are able to exhibit sweat responses directly after birth, most pronounced on the forehead [55,56]. Tsuzuki-Hayakawa et al. [57] compared the thermoregulatory responses to heat in children aged 9 months to 4.5 years to those of their mothers. They found that the average sweat rate per BSA was higher in young children (1.5 times higher). However, when looking at individual sweat glands, the output was about half that of the mothers. Furthermore, the sodium concentration in sweat was significantly lower in the children, which has been suggested to be due to more time for sodium reabsorption because of the slower sweat flow rates [18,57]. Sweat rates in children are also lower than in adults when measured relative to the rise in T_rec_ [35]. Another study by Tsuzuki [16], which compared the thermoregulatory response of age groups ranging from under 1 year to 8 years, found no significant differences in whole-body sweat rate per BSA across the different age groups.

Studies have also observed differences in the size of sweat glands between children and adults. Children generally have smaller sweat glands [58] and because the size of the sweat glands in adults is directly related to the sweat rate [59]; this difference has been suggested to explain the lower sweat rate observed in children. Moreover, local sweat rates across different body regions vary between children and adults, with children showing relatively higher sweat rates on the hands and feet, and adults on the back [60].

The overall lower sweat rate in children does not necessarily mean that their sweat response is inadequate or immature, as Falk and Dotan [22] state. Their review—which revisits some commonly accepted child–adult differences in thermoregulation—explains that maximal sweating rates, especially in children, have not been determined. This raises the question of whether children’s thermoregulation is limited by their sweating rates or if their lower sweating rate simply reflects a different thermoregulatory strategy. For instance, children may be more efficient in their sweating, with a greater proportion of their sweat output evaporating instead of dripping off the skin compared to adults. This is supported by Inbar et al. [35], who found that prepubertal boys had lower overall sweat rates than adults, yet demonstrated higher sweat efficiency—losing more heat through evaporation relative to the amount of sweat produced. The authors suggest this may be due to smaller sweat drops in children, which are less likely to coalesce and drip. Greater sweating efficiency may also be a result of the higher T_sk_ in children caused by greater dry heat gain in extreme heat, due to their larger surface area-to-mass ratio [22]. A higher T_sk_ is linked to a higher sweat temperature, which, in turn, facilitates evaporation. Moreover, lower sweat rates could slow down dehydration, enabling children to better withstand heat stress [22]. However, there is a lack of infant-specific data, leaving sweat efficiency and its role in managing heat stress in infants largely unexplored.

Thus, wet heat loss by sweating, together with dry heat loss, is an essential part of thermal control in infants directly after birth. The size of sweat glands in infants is even smaller than in children [58], and little is known about their actual sweating capacity or efficiency. Only one study has directly compared infant sweat rates to those of older children and found no significant differences in sweat rate per BSA across age groups [16]. How infants’ sweat rates compare to those of older individuals, and whether a lower rate indicates a limitation of their thermoregulatory system or a different strategy—accounting for their larger surface area-to-mass ratio, as suggested in children—requires additional research.

## 3. Infant Core Temperature During Heat Exposure

Few studies exist that have investigated how the differences described above are reflected in the ability to maintain a stable core temperature in infants during heat exposure. Tsuzuki et al. [57] compared mother–child pairs (children aged 9 months to 4.5 years) in a heat exposure study. The participants first spend 10 min in a thermoneutral room (T_a_ 25 °C, Rh 50%, V < 0.2 m/s), before being exposed to a hot room (T_a_ 35 °C, Rh 70%, V = 0.3 m/s) for 30 min, followed by another 30 min in the thermoneutral room. They found no significant differences in the relative change of T_sk_ between the mothers and their children. However, T_rec_ in children and infants significantly increased upon entering the hot room, while it remained stable in the mothers.

Under similar thermal conditions, Tsuzuki [16] investigated heat exposure in children of different age groups. Infants under the age of one showed significant increases in T_rec_ compared to children two years and older. There were no significant differences in the relative change of T_sk_ and whole-body sweat rate between the different age groups.

While both studies showed a more significant rise in T_rec_ in infants, indicating less stable core temperatures, it is difficult to determine how this rise relates to critical T_rec_ limits in children and infants. As argued by Falk and Dotan [22], ethical considerations prevent researchers from allowing participants to reach their physiological limit. Consequently, heat exposure ends before heat intolerance occurs, but rather once pre-determined T_rec_ thresholds are reached. These cut-off thresholds have often been based on adults. Applying them to infants may not be appropriate and might give them a definition-based disadvantage or advantage.

Given these limitations, experimental studies are unlikely to resolve how close observed increases in T_rec_ come to critical thermal limits in infants, highlighting the need for alternative approaches. Computational modeling—once a better understanding of infant thermoregulation in general is established—could simulate responses under varying thermal conditions. Additionally, wearable sensors could enable longer-term monitoring during naturally occurring heat events.

Since heat stroke—a severe form of heat-related illness—is defined not only by elevated core temperature but also by neurological dysfunction [61], assessing heat strain should extend beyond temperature measurements alone. In adults, early neurological signs such as deviations in walking patterns have proven useful in detecting the onset of heat stroke [62]. While comparable early markers have not yet been identified in infants, investigating mild deviations in neurological responses—particularly during natural heat exposure or heat-related hospital admissions—could offer a way to better understand heat vulnerability without experimental exposure to thermal limits.

## 4. Challenges of Researching Infant Thermoregulation

In addition to unclear T_rec_ limits, several factors complicate conducting research on infants’ thermoregulation. The first few months of life are characterized by rapid growth and developmental changes, resulting in significant variability in surface area-to-mass ratio among infants. T_rec_ also shows considerable variability over the first year of life [63]. The variability within this population becomes even more complex when taking into account other factors, such as sex, race, and ethnicity [64,65]. As a result, making broad generalizations about infants is challenging.

Further challenges include controlling the infant’s rest state. Active behaviors, such as crying, can alter an infant’s heart and metabolic rate, making it difficult to standardize conditions. Additionally, infants cannot be asked to perform controlled physical activities to reach a certain metabolic load, as is often done in research on thermoregulatory responses.

Moreover, tools and methods used to measure core temperature in adults are not always suitable for infants. T_rec_ has been commonly used to reflect core temperature; however, its measurement can be distressing for infants and upsetting for parents [66,67]. Tympanic thermometers are rapid and noninvasive, but require correct use, such as using the same ear in repeated measurements [68]. The Temple Touch Pro™ is a non-invasive monitor for core temperature that uses the heat flux technique and provides accurate readings in infants, but still requires further validation in extreme temperature ranges [69]. A balance must be struck between using validated, accurate methods while minimizing discomfort for the infant.

Ethical considerations are central to any study involving infants, especially as they cannot consent to participation and are completely reliant on caregiver protection. As a vulnerable population, infants must be protected from any procedures that may cause distress or harm. Ethical research design in this context requires measurement techniques that are minimal in invasiveness, careful management of infants’ exposure to heat, and continuous monitoring to avoid overheating or dehydration. Given the ethical constraints on studying heat stress in this population, future research should focus on study designs with minimal risks, such as those conducted in mild heat conditions, or consider alternative approaches, including computational models, infant thermal manikins, or naturally occurring heat events.

## 5. Maturity of Infant Thermoregulation and Future Research Directions

As outlined in the sections above, there are key differences in infants compared to children and adults. Infants have a relatively large surface area-to-mass ratio, which facilitates heat dissipation when T_a_ is below T_sk_. However, when T_a_ exceeds T_sk_, this could lead to more heat absorption, although the extent of this effect remains unclear. Size-adjusted metabolic heat production is highest during infancy, peaking around 8–9 months, during which infants must dissipate more heat per unit surface area to maintain thermal balance [37]. Additionally, it is theorized that a higher proportion of infants’ blood volume is directed to the skin during heat exposure, which could be beneficial for dry heat loss when T_a_ is below T_sk_ but reduces central blood flow, potentially leading to symptoms of heat intolerance. However, no studies have specifically investigated this in infants. Infants have a lower sweat output per gland [57], with multiple hypotheses offering explanations, including immature sweat glands, more efficient sweating, or a strategy to conserve water and prevent dehydration [22].

Overall, these differences do not seem to stem from an underdeveloped or immature thermoregulatory system, but rather from infants’ large surface area-to-mass ratio and high energy expenditure related to growth. Their unique physiological characteristics, combined with a reliance on caregivers for behavioral thermoregulation, primarily shape their vulnerability in extreme heat. The maturity of the sweating mechanism, however, remains unclear. Further research is needed to better understand how all these aspects affect core temperature in different thermal conditions, and how these responses compare to those of children and adults.

Another important gap in current research is how infants acclimatize to heat. In adults, repeated exposure to heat leads to physiological changes, including improved fluid balance and cardiovascular stability that can reduce heat-related health risks [70]. Children seem to acclimatize more slowly than adults [29], yet it remains unknown if infants develop similar adaptations and at what rate. Understanding this could clarify whether the early days of heat pose a higher risk due to lack of acclimatization or if proactive acclimatization protocols could protect infants, as currently being explored in elderly populations [71]. Future research could assess physiological changes after short-term heat exposure to mild heat, either in controlled settings such as climatic chambers (e.g., [72]) or during natural heatwaves.

## 6. Recommendations for Protecting Infants from Heat-Related Health Risks

Mitigating heat-related health risks for infants requires a multi-level approach. Caregiver behavior plays an essential role in protecting infants from heat exposure. This includes ensuring adequate hydration, dressing infants in light, breathable clothing, providing shade and cool environments, and recognizing early signs of overheating. For example, caregivers should be mindful of carrying infants in a sling during heat, as this can limit the ability of dry heat exchange [73], and covering a stroller with a dry muslin cloth can raise temperatures inside, while the combination of a moist muslin cloth and a fan could reduce temperatures [74].

These caregiver actions must be supported by broader public health strategies. Health authorities and policymakers should include infant-specific guidance in national heat warning systems and public awareness campaigns. Such materials should clearly outline early signs of heat-related illness and dehydration in infants—such as excessive sweating, reduced urine output, few or no tears when crying, rapid breathing, and confusion [75,76]—as well as safe cooling strategies and when to seek medical help. Health professionals and educators also play a key role in disseminating evidence-based recommendations to caregivers, such as revisiting commonly given advice like dressing infants in “one extra layer” than adults [77]—which needs to be reconsidered in hot weather.

Additionally, it is important to recognize that heat may not only affect infants directly—through heat strain—but also indirectly by increasing the risk of respiratory allergies, such as asthma, potentially through oxidative stress and activation of the TRPV1 receptor pathway [78,79]. These indirect effects on infant health further highlight the need for additional research and public health strategies to protect infants from heat-related harm.

## 7. Conclusions

The mechanisms for thermoregulation are already present at birth in full-term infants. Key differences in infants compared to older children and adults include a large surface area-to-mass ratio, high size-adjusted metabolic rate, low sweat output per sweat gland, and potentially high skin blood flow. Most of these differences do not appear to result from an inherently immature thermoregulatory system, but rather from infants’ larger surface area-to-mass ratio and higher energy expenditure. Infants seem to have certain advantages in heat dissipation during moderate heat (T_a_ < T_sk_) but may face challenges during extreme heat (T_a_ > T_sk_). However, the overall impact of these mechanisms on heat tolerance in infants is not yet fully understood.

A crucial factor in mitigating heat-related health risks for infants is providing caregivers with appropriate education and support. While further research is needed to fully understand the complexities of infant thermoregulation, immediate action should be taken to protect infants during heat exposure. Such efforts are vital in reducing the risk of heat-related health complications and thereby the vulnerability of this population.

## Data Availability

Not applicable.

## Abbreviations

The following abbreviations are used in this manuscript:

BSA	Body surface area
BEE	Basal energy expenditure
T_a_	Ambient temperature
TEE	Total energy expenditure
T_rec_	Rectal temperature
T_sk_	Skin temperature

## References

[1] Basagaña X., Sartini C., Barrera-Gómez J., Dadvand P., Cunillera J., Ostro B., Sunyer J., Medina-Ramón M. (2011). Heat waves and cause-specific mortality at all ages. Epidemiology.

[2] Basu R., Ostro B.D. (2008). A Multicounty Analysis Identifying the Populations Vulnerable to Mortality Associated with High Ambient Temperature in California. Am. J. Epidemiol..

[3] Fouillet A., Rey G., Laurent F., Pavillon G., Bellec S., Guihenneuc-Jouyaux C., Clavel J., Jougla E., Hémon D. (2006). Excess mortality related to the August 2003 heat wave in France. Int. Arch. Occup. Environ. Health.

[4] Scovronick N., Sera F., Acquaotta F., Garzena D., Fratianni S., Wright C.Y., Gasparrini A. (2018). The association between ambient temperature and mortality in South Africa: A time-series analysis. Environ. Res..

[5] Geruso M., Spears D. (2018). Heat, Humidity, and Infant Mortality in the Developing World.

[6] Lakhoo D.P., Blake H.A., Chersich M.F., Nakstad B., Kovats S. (2022). The Effect of High and Low Ambient Temperature on Infant Health: A Systematic Review. Int. J. Environ. Res. Public Health.

[7] Mannan I., Choi Y., Coutinho A.J., Chowdhury A.I., Rahman S.M., Seraji H.R., Bari S., Shah R., Winch P.J., El Arifeen S. (2011). Vulnerability of Newborns to Environmental Factors: Findings from Community Based Surveillance Data in Bangladesh. Int. J. Environ. Res. Public Health.

[8] Teyton A., Ndovu A., Baer R.J., Bandoli G., Benmarhnia T. (2024). Disparities in the impact of heat wave definitions on emergency department visits during the first year of life among preterm and full-term infants in California. Environ. Res..

[9] Mangus C.W., Canares T.L. (2019). Heat-related illness in children in an era of extreme temperatures. Pediatr. Rev..

[10] Folkerts M.A., Gerrett N., Kingma B.R.M., Zuurbier M., Daanen H.A.M. (2020). Care provider assessment of thermal state of children in day-care centers. Build. Environ..

[11] Danks D.M., Webb D.W., Allen J. (1962). Heat Illness in Infants and Young Children. Br. Med. J..

[12] Null J. (2025). Heatstroke deaths of children in vehicles. Proceedings of the 105th AMS Annual Meeting.

[13] Périard J.D., Eijsvogels T.M.H., Daanen H.A.M. (2021). Exercise under heat stress: Thermoregulation, hydration, performance implications, and mitigation strategies. Physiol. Rev..

[14] Cramer M.N., Jay O. (2016). Biophysical aspects of human thermoregulation during heat stress. Auton. Neurosci..

[15] Tansey E.A., Johnson C.D. (2015). Recent advances in thermoregulation. Adv. Physiol. Educ..

[16] Tsuzuki K. (2023). Effects of heat exposure on the thermoregulatory responses of young children. J. Therm. Biol..

[17] Inoue Y., Kuwahara T., Araki T. (2004). Maturation- and Aging-related Changes in Heat Loss Effector Function. J. Physiol. Anthropol. Appl. Human. Sci..

[18] Williams M.L. (2021). Global warming, heat-related illnesses, and the dermatologist. Int. J. Women's Dermatol..

[19] Xu Z., Etzel R.A., Su H., Huang C., Guo Y., Tong S. (2012). Impact of ambient temperature on children's health: A systematic review. Environ. Res..

[20] Smith C.J. (2019). Pediatric Thermoregulation: Considerations in the Face of Global Climate Change. Nutrients.

[21] Singer D. (2021). Pediatric Hypothermia: An Ambiguous Issue. Int. J. Environ. Res. Public Health.

[22] Falk B., Dotan R. (2008). Children’s thermoregulation during exercise in the heat—A revisit. Appl. Physiol. Nutr. Metab..

[23] Seneviratne S.I., Zhang X., Adnan M., Badi W., Dereczynski C., Luca A.D., Ghosh S., Iskandar I., Kossin J., Lewis S. (2021). Weather and Climate Extreme Events in a Changing Climate.

[24] (1987). Mosteller, Simplified calculation of body surface area. N. Engl. J. Med..

[25] Sigurdsson T.S., Lindberg L. (2020). Six commonly used empirical body surface area formulas disagreed in young children undergoing corrective heart surgery. Acta Paediatr..

[26] Schloesser R.L., Lauff M., Buxmann H., Veit K., Fischer D., Allendorf A. (2011). Three-Dimensional Body Scanning: A New Method to Estimate Body Surface Area in Neonates. Neonatology.

[27] WHO Child Growth Standards. https://www.who.int/tools/child-growth-standards/standards.

[28] Verbraecken J., Van de Heyning P., De Backer W., Van Gaal L. (2006). Body surface area in normal-weight, overweight, and obese adults. A comparison study. Metabolism.

[29] Bar-Or O. (1980). Climate and the Exercising Child-A Review. Int. J. Sports Med..

[30] Davies C.T.M. (1981). Thermal responses to exercise in children. Ergonomics.

[31] Taylor K.M., Giersch G.E.W., Caldwell A.R., Epstein Y., Charkoudian N. (2024). Relation of body surface area-to-mass ratio to risk of exertional heat stroke in healthy men and women. J. Appl. Physiol..

[32] Akavian I., Epstein Y., Rabotin A., Peretz S., Charkoudian N., Ketko I. (2025). The Significance of Body Surface Area to Mass Ratio for Thermal Responses to a Standardized Exercise-Heat Stress Test. Med. Sci. Sports Exerc..

[33] Rowland T. (2008). Thermoregulation during exercise in the heat in children: Old concepts revisited. J. Appl. Physiol..

[34] Drinkwater B.L., Kupprat I.C., Denton J.E., Crist J.L., Horvath S.M. (1977). Response of prepubertal girls and college women to work in the heat. J. Appl. Physiol..

[35] Inbar O., Morris N., Epstein Y., Gass G. (2004). Comparison of thermoregulatory responses to exercise in dry heat among prepubertal boys, young adults and older males. Exp. Physiol..

[36] Delamarche P., Bittel J., Lacour J.R., Flandrois R. (1990). Thermoregulation at rest and during exercise in prepubertal boys. Eur. J. Appl. Physiol. Occup. Physiol..

[37] Pontzer H., Yamada Y., Sagayama H., Ainslie P.N., Andersen L.F., Anderson L.J., Arab L., Baddou I., Bedu-Addo K., Blaak E.E. (2021). Daily energy expenditure through the human life course. Science.

[38] Hsu A., Heshka S., Janumala I., Song M.Y., Horlick M., Krasnow N., Gallagher D. (2003). Larger mass of high-metabolic-rate organs does not explain higher resting energy expenditure in children. Am. J. Clin. Nutr..

[39] Bauer J., Werner C., Gerss J. (2009). Metabolic rate analysis of healthy preterm and full-term infants during the first weeks of life. Am. J. Clin. Nutr..

[40] Azaz Y., Fleming P.J., Levine M., McCabe R., Stewart A., Johnson P. (1992). The Relationship between Environmental Temperature, Metabolic Rate, Sleep State, and Evaporative Water Loss in Infants from Birth to Three Months. Pediatr. Res..

[41] Wong B.J., and Hollowed C.G. (2017). Current concepts of active vasodilation in human skin. Temperature.

[42] Crandall C.G., Wilson T.E. (2015). Human cardiovascular responses to passive heat stress. Compr. Physiol..

[43] Beinder E., Trojan A., Bucher H., Huch A., Huch R. (1994). Control of skin blood flow in pre-and full-term infants. Neonatology.

[44] Jahnukainen T., van Ravenswaaij-Arts C., Jalonen J., Välimäki I. (1993). Dynamics of vasomotor thermoregulation of the skin in term and preterm neonates. Early Human. Dev..

[45] Takayanagi T., Fukuda M., Nakazawa M., Tanaka S., Yoshinaga M. (1999). Response of skin blood volume, velocity and flow to local warming in newborns, measured by laser Doppler flowmetry. Pediatr. Int..

[46] Ostchega Y., Porter K.S., Hughes J., Dillon C.F., Nwankwo T. (2011). Resting Pulse Rate Reference Data for Children, Adolescents, and Adults: United States, 1999-2008.

[47] Iliff A., Lee V.A. (1952). Pulse Rate, Respiratory Rate, and Body Temperature of Children between Two Months and Eighteen Years of Age. Child. Dev..

[48] Crandall C.G., González-Alonso J. (2010). Cardiovascular function in the heat-stressed human. Acta Physiol..

[49] Kirschen G.W., Singer D.D., Thode H.C., Singer A.J. (2020). Relationship between body temperature and heart rate in adults and children: A local and national study. Am. J. Emerg. Med..

[50] Heal C., Harvey A., Brown S., Rowland A.G., Roland D. (2022). The association between temperature, heart rate, and respiratory rate in children aged under 16 years attending urgent and emergency care settings. Eur. J. Emerg. Med..

[51] Martin H.L., Loomis J.L., Kenney W.L. (1995). Maximal skin vascular conductance in subjects aged 5-85 yr. J. Appl. Physiol..

[52] Falk B., Bar-Or O., MacDougall J.D. (1992). Thermoregulatory responses of pre-, mid-, and late-pubertal boys to exercise in dry heat. Med. Sci. Sports Exerc..

[53] Sato K., Kang W.H., Saga K., Sato K.T. (1989). Biology of sweat glands and their disorders. I. Normal sweat gland function. J. Am. Acad. Dermatol..

[54] Mancini A.J., Lawley L.P. (2014). Structure and function of newborn skin. Neonatal and Infant Dermatology E-Book.

[55] Foster K., Hey E., Katz G. (1969). The response of the sweat glands of the new-born baby to thermal stimuli and to intradermal acetylcholine. J. Physiol..

[56] Rutter N., Hull D. (1979). Response of term babies to a warm environment. Arch. Dis. Child..

[57] Tsuzuki-Hayakawa K., Tochihara Y., Ohnaka T. (1995). Thermoregulation during heat exposure of young children compared to their mothers. Eur. J. Appl. Physiol. Occup. Physiol..

[58] Landing B.H., Wells T.R. (1969). Sweat gland anatomy in genetic diseases. J. Chronic Dis..

[59] Sato K., Sato F. (1983). Individual variations in structure and function of human eccrine sweat gland. Am. J. Physiol.-Regul. Integr. Comp. Physiol..

[60] Arlegui L., Smallcombe J.W., Fournet D., Tolfrey K., Havenith G. (2021). Body mapping of sweating patterns of pre-pubertal children during intermittent exercise in a warm environment. Eur. J. Appl. Physiol..

[61] Yeo T.P. (2004). Heat Stroke: A Comprehensive Review. AACN Adv. Crit. Care.

[62] Buller M., Fellin R., Bursey M., Galer M., Atkinson E., Beidleman B.A., Marcello M.J., Driver K., Mesite T., Seay J. (2022). Gait instability and estimated core temperature predict exertional heat stroke. Br. J. Sports Med..

[63] Bayley N., Stolz H.R. (1937). Maturational Changes in Rectal Temperatures of 61 Infants from 1 to 36 Months. Child. Dev..

[64] Garcia-Souto M.D.P., Dabnichki P. (2016). Core and local skin temperature: 3–24 months old toddlers and comparison to adults. Build. Environ..

[65] Manna M.I.L.T., Dugasb J.P. (2008). Ethnicity and temperature regulation. Thermoregul. Human. Perform..

[66] Maxton F.J.C., Justin L., Gillies D. (2004). Estimating core temperature in infants and children after cardiac surgery: A comparison of six methods. J. Adv. Nurs..

[67] Kai J. (1993). Parents' perceptions of taking babies' rectal temperature. Br. Med. J..

[68] Dew P.L. (2006). Is tympanic membrane thermometry the best method for recording temperature in children?. J. Child. Health Care.

[69] Fujii T., Takakura M., Taniguchi T., Nishiwaki K. (2024). Accuracy of non-invasive core temperature monitoring in infant and toddler patients: A prospective observational study. J. Anesth..

[70] Périard J.D., Racinais S., Sawka M.N. (2015). Adaptations and mechanisms of human heat acclimation: Applications for competitive athletes and sports. Scand. J. Med. Sci. Sports.

[71] Cole E., Donnan K.J., Simpson A.J., Garrett A.T. (2023). Short-term heat acclimation protocols for an aging population: Systematic review. PLoS ONE.

[72] Pallubinsky H., Schellen L., Kingma B., Dautzenberg B., Van Baak M., van Marken Lichtenbelt W. (2017). Thermophysiological adaptations to passive mild heat acclimation. Temperature.

[73] Filingeri D., Cowley H., Merrick C., Gang P.S., Filingeri V.L. (2020). The effects of clothing layers on the thermoregulatory responses to short duration babywearing in babies under 12 months old. Physiol. Rep..

[74] Bin Maideen M.F., Jay O., Bongers C., Nanan R., Smallcombe J.W. (2023). Optimal low-cost cooling strategies for infant strollers during hot weather. Ergonomics.

[75] British Red Cross Learn First Aid for a Child Who May Have Heat Exhaustion. https://www.redcross.org.uk/first-aid/learn-first-aid-for-babies-and-children/heat-exhaustion.

[76] NSW Health Babies and Young Children in Hot Weather. https://www.health.nsw.gov.au/environment/beattheheat/Pages/babies-children-hot-weather.aspx.

[77] American Academy of Pediatrics Tips for Dressing Your Baby. https://www.healthychildren.org/English/ages-stages/baby/diapers-clothing/Pages/Dressing-Your-Newborn.aspx.

[78] Niu Z., Fang X., Sun J., Du S., Tang H., Shao W., Chen T., Nie J., Yu Z., Yao X. (2025). Exposure to Anthropogenic Heat Was Associated with the Increased Risks of Respiratory Allergy Symptoms in Preschool Children: A New Insight into the Urban Heat Island Effect. Environ. Sci. Technol..

[79] Deng L., Chen X., Ma P., Wu Y., Okoye C.O., Du D., Deng Q. (2024). The combined effect of oxidative stress and TRPV1 on temperature-induced asthma: Evidence in a mouse model. Environ. Pollut..

